# Influence of Manufacturing Process on the Conductivity of Material Extrusion Components: A Comparison between Filament- and Granule-Based Processes

**DOI:** 10.3390/polym16081134

**Published:** 2024-04-18

**Authors:** Maximilian Nowka, Karl Hilbig, Lukas Schulze, Timo Heller, Marijn Goutier, Thomas Vietor

**Affiliations:** Institute for Engineering Design, Technische Universität Braunschweig, Hermann-Blenk-Str. 42, 38108 Brausnchweig, Germany; k.hilbig@tu-braunschweig.de (K.H.); lukas.schulze@tu-braunschweig.de (L.S.); m.goutier@tu-braunschweig.de (M.G.); t.vietor@tu-braunschweig.de (T.V.)

**Keywords:** additive manufacturing, fused deposition modeling, material extrusion, 3D printing, electrically conductive, conductive polymer composite, filament extrusion, granule extrusion, electrical resistivity, functional material

## Abstract

The additive manufacturing of components using material extrusion (MEX) enables the integration of several materials into one component, including functional structures such as electrically conductive structures. This study investigated the influence of the selected additive MEX process on the resistivity of MEX structures. Specimens were produced from filaments and granules of an electrically conductive PLA and filled with carbon nanotubes and carbon black. Specimens were produced with a full-factorial variation of the input variables: extrusion temperature, deposition speed, and production process. The resistivity of the specimens was determined by four-wire measurement. Analysis of the obtained data showed that only the extrusion temperature had a significant influence on the resistivity of the MEX specimens. Furthermore, the impact of the nozzle diameter was evaluated by comparing the results of this study with those of a previous study, with an otherwise equal experimental setup. The nozzle diameter had a significant influence on resistivity and a larger nozzle diameter reduced the mean variance by an order of magnitude. The resistivity was lower for most process parameter sets. As the manufacturing process had no significant influence on the resistivity of MEX structures, it can be selected based on other criteria, e.g., the cost of feedstock.

## 1. Introduction

Additive manufacturing (AM), commonly known as 3D printing, offers new possibilities for product development. Despite being more expensive, layer-by-layer manufacturing has advantages over subtractive processes. The design flexibility is almost unlimited, as the complexity of the shape does not significantly impact the process cost. For example, undercuts or geometrically defined lattice structures can be used in the design process. Additive manufacturing processes offer a high degree of material freedom. Depending on the process, this can be used for multi-material part design.

One of the most widely used additive manufacturing processes is thermal material extrusion (MEX) [1,2,3]. The MEX processes differ both in terms of the feedstock (granules, plastic filaments) and the plasticization process [1,3]. The properties of the resulting components are influenced by the anisotropic microstructure, material selection, manufacturing process parameters, and MEX process parameters [1,3].

It has been shown that the process parameters used to produce filaments significantly affect the conductivity of both the filaments and the subsequently manufactured MEX structures [4].

**Figure 1:**
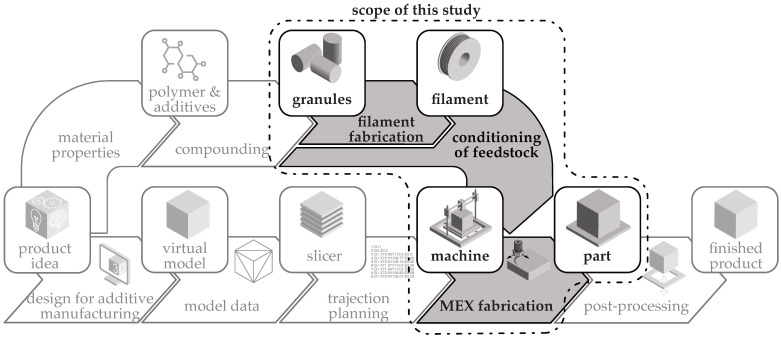
Scope of this study in the general additive manufacturing process chain, with additional material processing following [4].

Because of the similarities between the manufacturing processes of filaments and additive manufacturing using granulate MEX, such as the feeding of material through a screw, it was expected that the process variation would influence the resistivity. This study investigated the influence of the choice of additive manufacturing process, specifically filament-based material extrusion and granule-based material extrusion, on the resistivity of electrically conductive structures made with the material ALFAOHM (FILOALFA^®^ by Ciceri de Model Srl., Turin, Italy, abbreviated to FILOALFA^®^). Figure 1 shows the general process chain for additive manufacturing processes, supplemented by a materials engineering section. The section analysed in this study is highlighted.

### 1.1. Material Extrusion

Additive manufacturing processes use energy or binders to create parts with a defined shape from formless materials such as liquids, granules, powders, or filaments [5]. According to DIN 8580, these processes are classified as primary forming processes [6]. The material extrusion process used in this study is referred to as MEX-TRB/P, in accordance with DIN EN ISO 59200 [1,2,5,7]. The process was formerly known as fused deposition modelling (FDM), fused filament fabrication (FFF), or fused layer manufacturing (FLM) [2,3,7]. This method involves the material extrusion (MEX) of thermoplastic polymer (P). A thermal reaction (TRB) is used to bond the layers [3,5].

Various system technologies are available for the MEX process to accommodate a variety of feedstock. Thermoplastics are usually conveyed through continuous filament feeding or, in the case of granules, by a screw. Less common variants include the use of a plunger to convey pellets [1,3].

Regardless of the equipment used, the material application process is identical. The polymer is plasticized by heating and then pushed out of the nozzle, which is positioned one layer height away from the component [1,2,3,8]. The newly extruded strands are fused directly to the adjacent or underlying strands by the remelting of the surface of the already solidified strands [1,7]. A layer is formed by depositing multiple strands side by side [1,2]. The anisotropic properties of the component are a result of both the strand-by-strand structure of the layer and the layer-by-layer structure of the component. The anisotropy is especially noticeable in the electrical conductivity of conductive thermoplastics and in the mechanical strength characteristics [1,2,3]. The properties, such as conductivity, can be influenced by the equipment technology and type of feedstock used. For example, filaments undergo an additional extrusion step that affects the properties of the feedstock [1,3]. Furthermore, the extruder’s feeding principle can influence material properties through homogenization, degradation, etc. [9].

### 1.2. MEX Process Chain Overview

**Figure 2:**
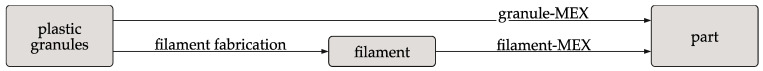
Processing routes for additive manufacturing a component from polymer granules.

The ISO 52900 standard does not address the different types of feedstock and system technologies required for processing [5]. Therefore, the terms ‘filament MEX’ and ‘granule MEX’ are used below to distinguish between the feedstocks used. Figure 2 illustrates the two process routes for processing granules into a finished product. The gray boxes represent the states, and the arrows show the processes that enable the changes.

The plastic granules can be processed directly into a part using a pellet extruder [1]. However, it is more common to use filaments as a feedstock. An upstream extrusion process is then required to produce filaments from the granules.

#### 1.2.1. Process Chain: Granule MEX

The granule MEX process is based on the extrusion of a granular feedstock. Granules are fed from a hopper through the extruder screw and into the heated barrel of the extruder. A rising temperature profile is created from the feed zone to the die by dividing the barrel into several heating zones. The screw, depending on its design, can assist in the feed of the material, the homogenization, and the heat input through the screw profile. The screw profile creates pressure in the melt, which compels the polymer melt to exit the die. Granule extrusion is especially appropriate for high application rates because of the process’s nature, making it well-suited for large-format components [1,3,9].

#### 1.2.2. Process Chain: Filament MEX

The filament MEX process uses a feedstock in the form of a wire, known as a filament. A continuous extrusion process is used to produce the filament from plastic granules. Filament extruders are designed as ram extruders. In these extruders, the filament is fed into the heated nozzle by a feed mechanism, usually in the form of a coarse wheel. The viscous melt is pushed from the nozzle orifice by the continuous feeding of the filament. The geometric quality of the filament is critical to the reliability of the process, as the control assumes a constant diameter for the filament. Any deviation leads to an incorrect amount of material being extruded [1,3,8].

### 1.3. Current Literature

Previous publications on additive processing of electrically conducting polymer composites have focused on the influence of AM process parameters on resistivity. The studies differ fundamentally in terms of the process variants that are used. The majority of publications use the more common process of filament MEX [10,11] (the list is a selection). Granule MEX is much less commonly used to produce electrically conducting specimens [12,13]. Table 1 provides a summary of relevant publications in this field, comparing the materials used, different MEX parameters, and characterization methods.

The resistivity of the filament MEX process has been extensively studied with respect to the influence of most process parameters [10,11]. Studies have often used commercially available filaments [4,10,11,18,20]. In contrast, the granule MEX process has only been used to process custom-made conductive polymer composites. The process parameters and their influence on the resistivity of components manufactured by granule MEX have not been investigated. Furthermore, self-developed or unspecified granule extruders are often used [12,16]. Only Georgopoulou et al. (2022) have used a commercially available system [13]. In a single study, the resistivity of the fabricated MEX test specimens was determined using a four-wire measurement, but no contact agent was used to reduce the measurement error [12]. Due to these vast differences in approaches, materials, MEX machines, and measurement methods, a direct comparison of the two MEX methods has not been possible to date.

This paper therefore examines whether variations in process parameters that are known to cause resistance changes in filament MEX also affect resistance changes in screw-based granule MEX. In addition, we analyzed whether the granule MEX process could achieve better conductivities than filament MEX, due to the elimination of the filament production step.

A direct comparison between granule MEX and filament MEX was made using the same commercial feedstock in granule and filament forms. To support the reproducibility of this experiment, only commercially available AM machines were used. The design of experiments (DoE) investigated the MEX process parameters of process chain variation, extrusion temperature, and deposition speed. The resistivity was characterized on MEX specimens produced using both manufacturing processes.

## 2. Materials and Methods

**Figure 3:**
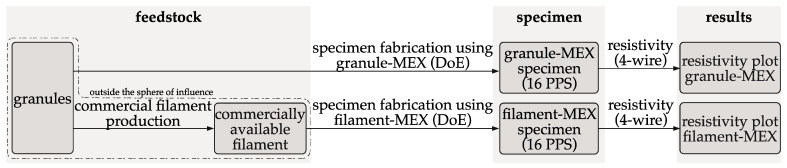
Schematic overview of the phases in this study of the specimen fabrication and characterization (PPS = process parameter set).

This study examined how the electrical conductivity of additively manufactured structures was affected by MEX processing methods, specifically granule MEX and filament MEX. Figure 3 provides an overview of the two methods used for specimen fabrication with MEX and the electrical characterization.

A brief summary of the research approach is listed below. In the following paragraphs, each item is presented in greater detail.

- MEX specimens were manufactured from filaments and granules using two feedstock-specific AM machines (filament MEX and granule MEX).
- MEX specimens were produced for each process using 16 different process parameter sets (PPSs).
- The specimens’ resistivity was determined using a four-wire measurement method in accordance with DIN EN ISO 3915:2022-5 [5].

### 2.1. Materials

**Figure 4:**
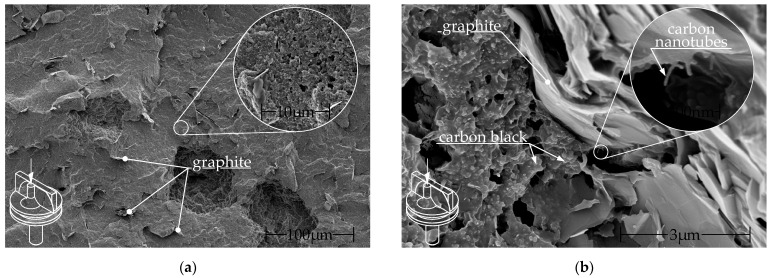
SEM images of the perpendicular fracture surface of commercial Alfaohm filament: (**a**) low magnification: graphite particles visible in the fracture surface and (**b**) high magnification: graphite particles as well as carbon black and carbon nanotubes visible.

The study used commercially sourced granules and filaments from FILOALFA^®^. This compound consists of PLA filled with CNTs, as stated by the manufacturer, along with undisclosed amounts of carbon black and graphite [4,23,24]. Contreras-Naranjo et al. discovered a fraction of MWCNT of approximately 3% of weight, with a CNT/CB ratio of 1:10 [24]. The filament is produced from the same commercially available granules. Figure 4 shows SEM images of the filament surface at two different magnifications.

The specimens were cryo-fractured in liquid nitrogen and sputtered with a 4 nm thick platinum layer. Images were taken with a Helios G4 CX (Field Electron and Ion Company (FEI), Hillsboro, OR, USA) using the secondary electron detector at 3 to 5 keV. The fracture surface was perpendicular to the filament and thus to the extrusion direction (out of the image plane). In both Figure 4a,b graphite particles can be seen. The graphite particles are oriented along the extrusion direction. The conductive additives carbon black and CNTs are visible in Figure 4b.

The process parameters for the commercial filament production were unknown and could not be influenced. The manufacturer’s recommended processing temperatures are between 190–210 °C, with deposition speeds of 10–50 mm/s and build platform temperatures of 0–50 °C. According to the manufacturer, a resistivity (measured according to ASTM D 257 [25]) of 15 Ωcm within a layer (no infill angle specified) and 20 Ωcm perpendicular to the layer can be expected within this process range [23]. There is no formal standard to categorize materials based on conductivity. Instead, the rating is based on literature values. Electrically conductive polymers with a resistivity greater than 10^12^ Ωcm are classified as electrical insulators. In the range of 10^10^ to 10^6^ Ωcm, plastics are classified as electrostatically dissipative. From 10^6^ Ωcm to 10^3^ Ωcm, plastics are dissipative. Plastics below 10^3^ Ωcm are classified as electrically conductive, which includes Alfaohm with a resistivity of 20 Ωcm [26,27,28].

All electrical contact surfaces are pre-treated with colloidal silver ink EMS#12640 (Electron Microscopy Sciences, Hatfield, PA, USA), as explained in further detail in Section 2.3.

### 2.2. Additive Manufacturing Machines

The test specimens were additively manufactured using commercially available equipment. A Toolchanger^®^ (E3D-Online, Chalgrove, Oxfordshire, UK) was used to fabricate the specimens from filament. An NX Pro Pellets-Tumaker^®^ (INDART3D, Irun, Gipuzkoa, Spain) is used to fabricate the specimens directly from the granules.

**Figure 5:**
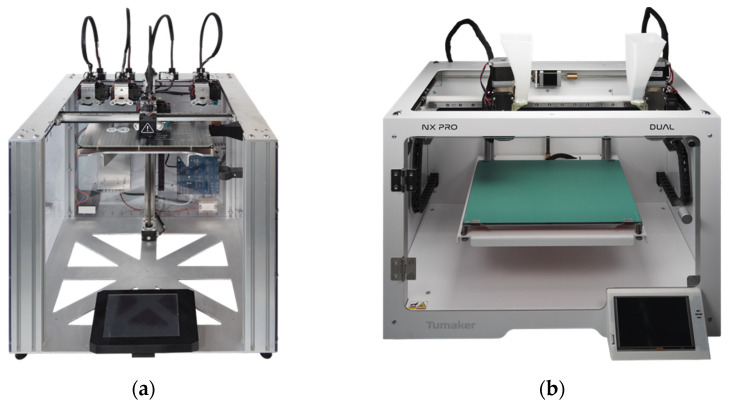
Additive manufacturing machine used for specimen fabrication: (**a**) E3D-Toolchanger for fabricating specimens using filament MEX and (**b**) NX Pro Pellets-Tumaker^®^ to produce specimen using granule MEX.

The E3D toolchanger, as shown in Figure 5a, was fitted with a Hemera direct drive filament extruder (E3D-Online, Chalgrove, Oxfordshire, UK), suitable for the filament with diameter of 1.75 mm that was used for sample production. The extruder was fitted with a hardened and coated 800 µm diameter Nozzle X (E3D-Online, Chalgrove, Oxfordshire, UK).

The NX Pro Pellets-Tumaker granule extruder, as shown in Figure 5b, features two vertically aligned miniature screw extruders for extruding granules. Two independent heatable zones in each extruder provide temperature control [29]. The screw has a diameter of 8 mm, an L/D ratio of 7.5, and a compression ratio of 1.7:1. The system manufacturer supplies nozzles with a diameter ranging from 400 to 800 µm, the latter was used during this study.

### 2.3. Resistivity Measurement

There currently exists no standard specifically for determining the resistivity of MEX feedstock, or electrically conductive structures additively manufactured from it. Therefore, resistivity measurements were carried out in accordance with ISO 10350-1 [30], based on DIN EN ISO 3915:2022-5 [31]. This standard applies to electrically conductive plastics with an isotropic resistivity of less than 10^6^ Ωcm [31].

MEX structures exhibit anisotropic electrical properties due to the strand-by-strand and layer-by-layer nature of the manufacturing process. Therefore, the measurement method specified in DIN EN ISO 3915:2022-5 was modified, as described by Nowka et al. [4,31]. The thickness of the sample was limited to one layer and its length was shortened to 60 mm to fit onto a microscope slide, preventing deformation during handling. Due to the reduced length, the distances between the electrodes were altered. Nevertheless, the high conductivity of the material ensured that ISO 3915:2002-5 was still valid within its range [4,31]. For each specimen, the thickness was measured at three points using a QuantuMike^®^ 293-140-30 micrometer screw (Mitutoyo Corporation, Kawasaki, Japan). Specimens with a thickness deviation of more than 10% from the nominal dimension at a single measuring point were rejected [31]. For each rejected specimen, a new specimen was produced and remeasured.

**Figure 6:**
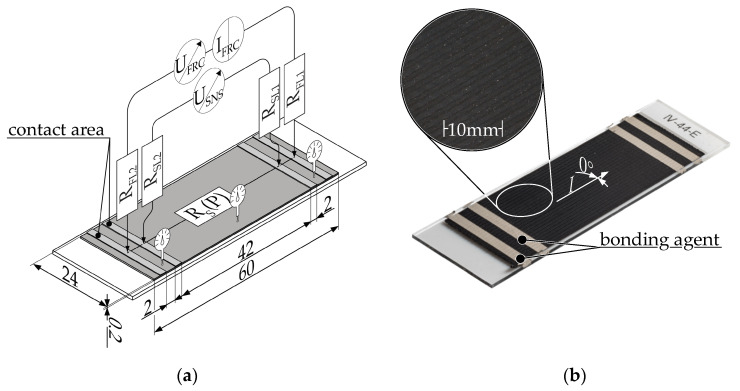
Measurement setup to determine the resistivity of MEX specimens by using 4-wire measurement: (**a**) Schematic illustration and wiring of the experimental setup for MEX specimens. R_S_(P) = resistance of the sample depending on the MEX parameters, R_FL_= resistance force lead, R_SL_ = resistance sense lead, I_FRC_ = forced current, U_FRC_ = voltage needed to force current, U_SNS_ = measured voltage drop across specimen [4]. (**b**) Granule MEX specimen with applied electrical bonding agent.

Figure 6 shows the schematic representation of the measurement setup, including the sample geometry and circuit as well as a real specimen.

The MEX specimens were fabricated on a microscope slide in a single layer, with a fill pattern orientation of 0° in the direction of current flow. To reduce contact resistance, two 2 mm wide strips of EMS#12640 silver paste were applied to each end of the sample. Colloidal silver paste is one of most used techniques to reduce the contact resistance of additively manufactured conductive structures [4,10,32,33,34,35,36,37,38]. The voltage drop was measured with a Keithley 2460 Sourcemeter (Keithley Instruments, Solon, OH, USA) via the two inner measuring contacts at a distance of 42 mm. The 100 µA measurement current was sourced via the outer contacts, which were located 2 mm from the measurement contacts.

According to Ohm’s law, the electrical resistance R can be calculated from the measured current *I* and the voltage drop *U*:

(1) $$R=\frac{U}{I}$$

In the context of MEX, the process-parameter-dependent resistivity $\rho_{P}$ can be calculated from the geometric dimensions length l and cross section A as well as the process-parameter-dependent resistivity $R_{P}$:

(2) $$\rho_{P}=R_{P}\cdot \frac{A}{l}$$

The cross-sectional area *A* was calculated using the average of the three thickness measurements points.

### 2.4. Design of Experiments

This study contributes to the previously identified need for research by investigating the influence of process choice and process parameters in granule MEX and filament MEX on specimen resistivity.

Prior to the determination of the design of experiments (DoE), preliminary tests were carried out by varying the process parameters frequently investigated in literature, such as layer height, deposition speed, extrusion temperature, and nozzle diameter. The filament MEX process allows for processing at temperatures ranging from 180 °C to 220 °C, with deposition rates of up to 70 mm/s. It is possible to produce layers as thin as 100 µm using nozzle diameters of 400 µm and larger. In contrast, the granule MEX production system can only reliably produce ALFAOHM structures with a layer thickness of 200 µm and a nozzle diameter of 800 µm. As a result, a constant layer height of 200 µm and an 800 µm nozzle were used for all specimens in both process variants. The preliminary tests furthermore indicated that the granule MEX system was unable to dispense the polymer melt reliably at temperatures below 190 °C, due to its high viscosity. Additionally, at temperatures above 220 °C, the granules soften in the feed zone due to heat conduction through the screw and heat convection, which also hinders reliable processing. As a result, the extrusion temperature input factor was limited to the range of 190–220 °C. Because the pellet extruder has two heating zones, a suitable temperature profile for the pellet MEX system was also identified in preliminary tests. Preliminary tests showed that reliable melt flow was only achievable when the temperature in the nozzle zone matched the temperature in the upstream compression zone. Using other settings, uneven extrusion occurred because the motor torque was insufficient to maintain a constant material flow.

Based on the results of the preliminary tests, a full factorial test plan was created for the production of MEX samples. Five specimens were produced for each parameter set. The temperature of the build platform was equal to the ambient temperature (22 °C). No cooling and no shells were used. Table 2 is a summary of the DoE and its input factors.

The design of experiments yielded 32 individual process parameter sets, each consisting of five specimens. This resulted in a total of 160 specimens.

The screw speed in the granule MEX process and the feed gear speed in the filament extruder were automatically determined by the slicing software and were therefore not input factors.

The influence of nozzle diameter has rarely been investigated, as shown in the table (literature review). Previous studies in this field have been conducted by Stankevich et al. (using PLA/CB and PVDF/G), Dembek et al. (using PLA/CNT), and Nassar et al. (using PCL/copper) [11,20,34]. The DoE in this study matched that of Nowka et al. in [4]. In that study, filament MEX samples were produced using a nozzle diameter of 400 µm, as opposed to 800 µm in this study, under otherwise identical conditions. A comparison can therefore be made to determine the influence of the nozzle diameter on resistivity.

## 3. Results and Discussion

This section presents and interprets the measurement results for the influence of the process choice (filament MEX/granule MEX) and the process parameters, extrusion temperature and deposition rate, on the resistivity.

### 3.1. Resistivity as a Function of Process Variant and Process Parameters

**Figure 7:**
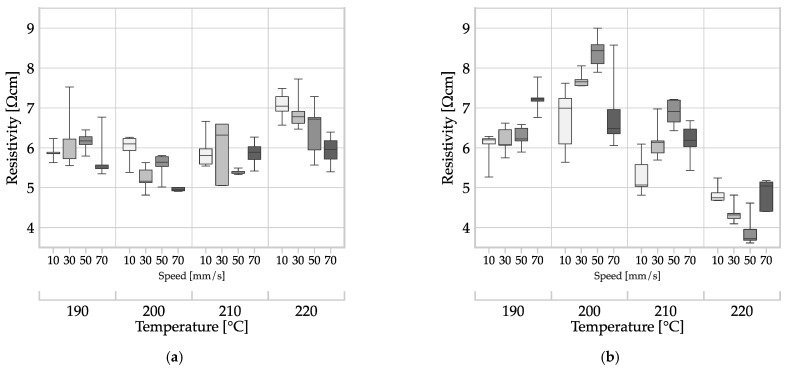
Effect of the input factors of deposition speed, temperature, and MEX variant on the resistivity of monolayers of ALFAOHM^®^ produced by MEX: (**a**) filament MEX and (**b**) granule MEX.

Figure 7 shows the results of the investigation into the influence of the MEX process variant on resistivity. The results are presented in two plots with the same scaling for resistivity on the *y*-axis, differing only in the MEX process used to produce the samples. Figure 7 also illustrates the influence of the MEX process parameters.

The measured values of filament MEX were close to the mean value of 5.94 Ωcm ± 0.67 Ωcm across all samples. The resistivity values of all filament MEX specimens was between a minimum of 4.81 Ωcm and a maximum of 7.72 Ωcm. In contrast, the average resistivity of the granule MEX specimens (see Figure 7b) was 6.11 Ωcm ± 1.26 Ωcm, with minimum and maximum values of 3.61 Ωcm and 9.00 Ωcm, respectively.

To investigate the impact of the process choice on resistivity, a one-way analysis of variance (ANOVA) was conducted using Minitab^®^ Version 21 (Minitab GmbH, Munich, Germany). The resistivity was set as the response, and the process (filament MEX, granule MEX) was the explanatory categorical variable, with a significance level of α = 0.05. The statistical analysis result of (*p* = 0.290 > α) indicates that the choice of process did not have a significant impact on the specific resistance. To identify the influence of process parameters within each of the two process variants, a linear regression model was fitted for the influences of temperature and speed, including second-order factors and interactions. The model was refined by backward elimination of terms (α to remove = 0.1). The results for these models are presented in Table 3.

The regression model for the filament MEX provided an inaccurate approximation with an R^2^ value of 51.50%. This suggests that some statistically significant influencing factors were not considered during the test planning and modelling. In contrast, the model for granule MEX demonstrated a significantly better approximation of the measurement data with an R^2^ value of 71.39%. For both methods, the R^2^ (predicted) values were only slightly below the R^2^ values, suggesting that overfitting was very unlikely.

**Figure 8:**
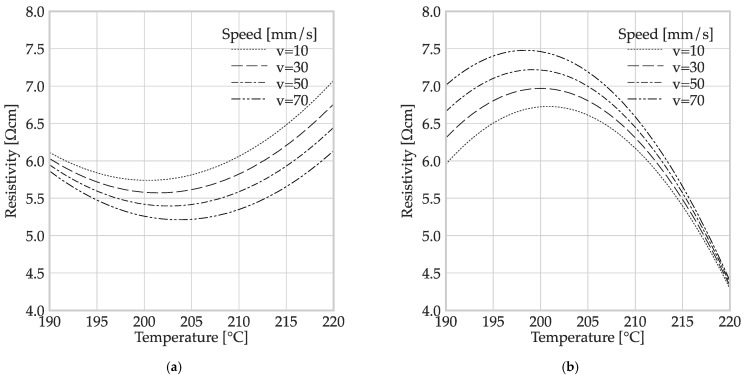
Plots of regression models for the influence of process parameters for different MEX processes: (**a**) regression model for filament MEX and (**b**) regression model for granule MEX.

The regression models indicated that temperature had a significant (P(temperature [°C]) = 0.000) and non-linear effect (P(temperature [°C] · temperature [°C]) = 0.000) in both MEX processes. However, the speed factor did not have a significant effect in any combination (*p* > α = 0.05). A graphical representation of the two regression models is presented in Figure 8.

Both graphs clearly show the non-linear influence of temperature due to the parabolic shape. The minor differences between the curves for the different speeds show the small (non-significant) influence of the deposition speed on the resistivity. The influence of the temperature was more noticeable for the granule MEX. The different curvature directions of the parables should be highlighted. The minimum specific resistance for filament MEX was reached at a temperature within the range of 200 °C, while specimens produced using granule MEX reached their maximum resistivity at the same temperature.

A possible explanation for the discrepancy in Figure 8 could be found in the different process chains. Figure 8 highlights that the polymer underwent a first extrusion process during filament production, after compounding the polymer composite. Nowka et al. have reported a significant influence of the filament manufacturing process parameters on the specific resistivity of the resulting MEX structures. Components made from commercially produced filaments exhibited significantly lower conductivity than those made from in-house-produced filaments from the same granules. In the granule MEX manufacturing process the granules are extruded only once after compounding, while in filament MEX, extrusion occurs twice before the part is finished. Thus, damage to the CB aggregates may occur during filament production, leading to degradation or breakage [4,26].

The conductivity may be more affected by the process parameters in granule MEX (refer to Figure 8b) because this is the first extrusion step the material undergoes. As temperature increases, the difference between the speed curves for granule MEX decreases. This is likely due to the decrease in viscosity, which reduces the effect of other factors.

The regression model does not consider the internal geometry of the nozzle as a potential input factor. The internal nozzle geometry can vary to accommodate different feedstocks (NozzleX/TUMaker Nozzle), even when the nozzle orifice diameter is identical. These geometries can impact the distribution and orientation of conductive fillers in the exiting melt, as well as the swelling behavior of the polymer at the exit, ultimately affecting electrical conductivity [39,40,41,42]. It is therefore possible that the different nozzle geometries influence the resistivity.

### 3.2. Influence of the Nozzle Diameter on Resistivity in Filament MEX

**Figure 9:**
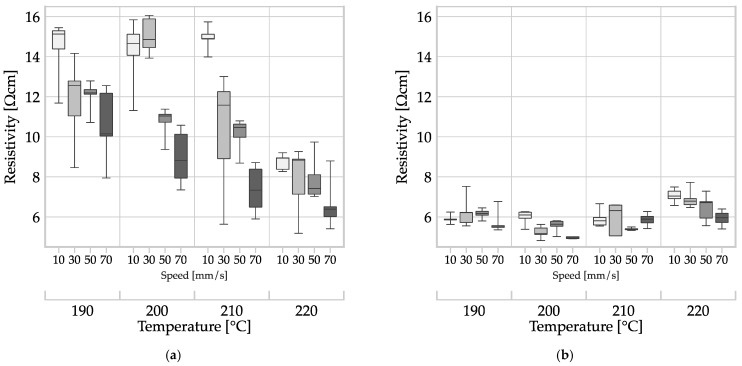
Influence of nozzle diameter on the resistivity of MEX structures manufactured by filament MEX: (**a**) 400 µm nozzle diameter—data from Nowka et al. [4] and (**b**) 800 µm nozzle diameter—data from this study.

The process parameter sets used for filament MEX in this study were identical to those of Nowka et al., except for the nozzle diameter which was 400 µm in the previous study [4]. Additionally, both studies used the same material, ALFAOHM, and methods for specimen preparation and resistivity measurement. By comparing the results of the two studies, it is possible to make a statement about the influence of the nozzle diameter (400 µm and 800 µm) on the resistivity of filament MEX structures. The results are shown in box plots in Figure 9.

The resistivity of specimens produced with the 400 µm nozzle (refer to Figure 9a) ranged from 16.06 Ωcm to 5.17 Ωcm, while the range for specimens produced with an 800 µm nozzle (refer to Figure 9b) was narrower, ranging from 7.72 Ωcm to 4.81 Ωcm. Both nozzle diameters led to a similar minimum. Moreover, the mean resistivity across all process parameter sets for the 800 µm nozzle at 5.94 Ωcm was lower than that of the 400 µm nozzle at 10.71 Ωcm. The variance of the samples produced with the 800 µm nozzle at 0.207 Ω^2^cm^2^ was an order of magnitude lower than that of the samples produced with the 400 µm nozzle at 2.211 Ω^2^cm^2^.

A single-factor ANOVA (α = 0.05) was used to compare the two data sets, indicating that the nozzle diameter had a significant effect (*p* = 0.000) on the resistivity of the components made with filament MEX.

Stankevich et al. observed a decrease in resistivity as nozzle diameter increased for a CB-PLA composite [11]. The observed behavior may have been due to the smaller nozzle’s increased susceptibility to clogging, or a restricted flow cross-section, resulting in uneven polymer melt application and higher variability. Alternatively, the higher thermal energy of the volume flow from the 800 µm nozzle may have enhanced the bonding of the deposited strand to the surrounding strands. The specimens were standardized to a width of 24 mm. As a result, specimens produced with the 800 µm nozzle had half the number of traces compared to those produced with the 400 µm nozzle. This reduction in contact areas between strands reduces the risk of manufacturing defects and mitigates the negative impact of the boundary layers of the strands. According to literature, structures with thicker strands are more conductive [22].

## 4. Summary and Conclusions

The aim of this study was to compare the influence of different MEX processing methods on the specific resistivity. Filament MEX and granule MEX were used as examples. The main results of processing ALFAOHM^®^ composites with different MEX methods are as follows:

1. The resistivity of electrically conductive MEX structures was not significantly affected by the processes investigated.
2. Better absolute lowest resistivity could be achieved with the granule MEX process using the optimal process parameter set.
3. The nozzle diameter had a significant influence on the resistivity, with smaller diameters leading to significantly higher resistivity.

As part of the investigation, a set of robust process parameters for the fabrication of specimens by MEX had been identified through preliminary testing. These tests revealed that reliable processing was only achievable for both MEX variants from a temperature of at least 190 °C and a layer thickness of at least 200 µm. Based on this, 32 sets of process parameters were derived by varying the MEX process, extrusion temperature, and deposition speed. Following the design of experiments, specimens were produced using both filament and granule MEX with the geometries as proposed by Nowka et al [4]. Despite the preliminary findings, producing geometrically accurate specimens with granule MEX and the additive manufacturing system NX Pro Pellets proved to be challenging. The rejection rate of these specimens was an order of magnitude higher than for filament MEX. The specific resistance of the samples was determined in accordance with ISO 3915 specifications [31]. Analysis of the investigated process parameters showed that the resistivity of both MEX processes was only statistically affected by extrusion temperature.

Comparing the MEX results of this study with those of Nowka et al. revealed a significant influence of nozzle diameter on resistivity [4]. The findings show that larger nozzle diameters can improve conductivity.

The findings in this work serve to enable the integration of conductive structures into multi-material part design. This requires extensive knowledge in areas such as material selection, process parameters, path planning, electrical contacting, and design for AM. Based on the results of this study, the following recommendations can be provided for the design and manufacturing of products using MEX:

- Larger nozzle diameters enable both a higher material deposition rate and, depending on the chosen process parameter set, a better overall conductivity. Therefore, if the geometric constraints, such as the thinnest wall of the structure, allow, larger nozzle diameters are preferable.
- The granule MEX process can directly process granules and is more economical due to the elimination of filament production. Therefore, it is preferable to filament extrusion. However, precise process control is required.

Future research should aim to close gaps in the existing knowledge, and further develop design guidelines. This study compared sample conductivity using an 800 µm nozzle with the results of Nowka et al., who used a 400 µm nozzle, and found a significant correlation between the conductivity of the ALFAOHM material and the nozzle diameter [4]. However, the analysis is based on only two discrete nozzle diameters. Therefore, it is necessary to investigate additional nozzle diameters to make a conclusive statement. A comprehensive data set regarding the influence of different nozzle diameters on resistivity is important, because it determines minimum xy-plane structure size and optimum layer height.

The influence of nozzle geometry on the resistivity of MEX structures has not been investigated yet. Structures made from composites with conductive fillers, such as CNTs or graphene with a high aspect ratio, can benefit from a suitable nozzle geometry selection.

## Figures and Tables

**Table 1:** Literature review of the fabrication of electrically conductive structures by granule and filament material extrusion from polymer composites containing carbon allotropes.

	Study	Kumar et al. [12]	Bajpai et al. [14,15]	Georgopoulou et al. [16]	Georgopoulou et al. [13]	Watschke et al. [10]	Sanchez et al. [17]	Gao and Meisel [18]	Zhang et al. [19]	Dembek et al. [20]	Yang et al. [21]	Paz et al. [22]	Nowka et al. [4]	Stankevich et al. [11]
Material and feedstock processing	Commercially available					◆		◆		◆			◆	◆
	Matrix polymer	EVA	EVA	TPE	TPE	PLA, PCL	PLA	PLA	ABS	PLA	PVA	ABS	PLA	PLA PVDF
	Fillers (legend below)	GR	G, CNT	CB	CB	CB, CNT, CP	GR, G	CB, GnP	CB	CNT	GnP	GnP	CB, CNT	G, CB
	Filament MEX					◆	◆	◆	◆	◆	◆	◆	◆	◆
	Granule MEX	◆	◆	◆	◆									
MEX parameters	Layer height							⊛	⊛	⊛		⊛	⊛	⊛
	Deposition speed					⊛		⊛					⊛	
	Extrusion temperature					⊛		⊛		⊛	⊛		⊛	
	Build platform temp.													
	Infill pattern						⊛					⊛		⊛
	Infill pattern orientation					⊛					⊛	⊛		
	Infill percentage								⊛					
	Line width								⊛			⊛		
	Nozzle diameter													⊛
	Flow rate					⊛				⊛				
	Cooling									⊛				
Characterization	Electrical bonding		Ag, Cu			Ag	Ag			Ag	Ag	Ag	Ag	
	Resistivity filament					◎	◉		◉			◎	◉	
	Resistivity MEX specimen	◉	◎		◎	◎	◉	◎	◉	◉	◎	◎	◉	◉
	SEM	◆	◆				◆		◆		◆		◆	◆

Matrix polymers: EVA = ethylen-vinylacetat-copolymer; TPE = thermoplastic elastomer; PLA = polylactic acid; PCL = polycaprolactone; ABS = acrylonitrile butadiene styrene; PVDF = polyvinylidene fluoride. Fillers: GR = graphite; G = graphene; CNT = carbon nanotube (no distinction between single and multiwall CNT); CB = carbon black; CP = copper particles; GnP = graphene nanoplatelets. Electrical bonding: Ag = silver paste/epoxy; Cu = copper paste/epoxy. ◆ = true statement; ⊛ = varied parameters. Resistance measurement: ◎ = 2-wire measurement; ◉ = 4-wire measurement.

**Table 2:** Overview of full factorial design of experiments.

DoE Input Factor	Lower Limit	Increment	Upper Limit
Extrusion temperature [°C]	190	10	220
Deposition speed [mm/s]	10	20	70
MEX manufacturing process	Filament MEX	-	Granule MEX

**Table 3:** Parameter results of the regression model for both process variants.

		Filament MEX	Granule MEX
Model	S [Ωcm]	0.482835	0.696061
	R^2^ [%]	51.50	71.39
	R^2^ (predicted) [%]	45.11	68.03
*p*-Value	Extr. temperature [°C]	0.000	0.000
	Deposition speed [mm/s]	0.125	0.068
	Extr. temperature [°C] · extr. temperature [°C]	0.000	0.000
	Extr. temperature [°C] · deposition speed [mm/s]	0.080	0.092

## Data Availability

The data presented in the study are openly available in FigShare at https://figshare.com/articles/dataset/dx_doi_org_10_6084_m9_figshare_25610775/25610775.
